# Screening of Duck Tembusu Virus NS3 Interacting Host Proteins and Identification of Its Specific Interplay Domains

**DOI:** 10.3390/v11080740

**Published:** 2019-08-12

**Authors:** Yawen Wang, Shuai Zhang, Yi Tang, Youxiang Diao

**Affiliations:** 1 College of Animal Science and Technology, Shandong Agricultural University, 61 Daizong Road, Tai’an 271018, China; 2 Shandong Provincial Key Laboratory of Animal Biotechnology and Disease Control and Prevention, Shandong Agricultural University, 61 Daizong Road, Tai’an 271018, China; 3 Shandong Provincial Engineering Technology Research Center of Animal Disease Control and Prevention, Shandong Agricultural University, 61 Daizong Road, Tai’an 271018, China

**Keywords:** DTMUV, yeast two-hybrid system, HELICc domains of NS3, yeast regressive verification, cellular co-localization, GST Pull-Down assay

## Abstract

NS3 protein is a member of the non-structural protein of duck Tembusu virus (DTMUV), which contains three domains, each of which has serine protease, nucleotide triphosphatase, and RNA helicase activities, respectively. It performs a variety of biological functions that are involved in the regulation of the viral life cycle and host immune response. Based on the yeast two-hybrid system, we successfully transformed pGBKT7-NS3 bait plasmid into Y2H Gold, tested it to prove that it has no self-activation and toxicity, and then hybridized it with the prey yeast strain of the duck embryo fibroblast cDNA library for screening. After high-stringency selection, positive alignment with the National Center for Biotechnology Information database revealed nine potential interactive proteins: MGST1, ERCC4, WIF1, WDR75, ACBD3, PRDX1, RPS7, ND5, and LDHA. The most interesting one (PRDX1) was selected to be verified with full-length NS3 protein and its three domains S7/DEXDc/HELICc using yeast regressive verification and GST Pull-Down assay. It denoted that PRDX1 does indeed interact with HELICc domains of NS3. NS3 is involved in the RNA uncoiling process of viral replication, which may cause mitochondrial overload to create oxidative stress (OS) during DTMUV attack. We deduced that the HELICc domain binding partner PRDX1, which regulates the p38/mitogen-activated protein kinase pathway (p38/MAPK) to avert OS, causing apoptosis, making it possible for viruses to escape host immune responses.

## 1. Introduction

Tembusu virus (TMUV) was first isolated from mosquitoes in Malaysia in 1955. Since 2010, the pathogen has widely spread in China’s laying ducks and was identified as the new duck TMUV (DTMUV) [1]. In recent years, the DTMUV epidemic has indeed brought considerable economic losses to the duck industry [2]. Members of flaviviruses mostly cause mild febrile illness, hemorrhagic fever [3], encephalitis [4], and shock syndrome in humans and animals [5]. Typically, ducks infected with DTMUV show symptoms of reduced egg production, severe neurologic disorders, and hemorrhagic ovaritis, which are slightly different from those of other flavivirus-infected hosts [6].

The DTMUV genome is composed of three structural proteins (C, prM, and E) [7], which mediate virus attachment, entry, and encapsidation [8]. Virus non-structural proteins (NS1, NS2A, NS2B, NS3, NS4A, NS4B, and NS5) [9] modulate viral replication and transcription and attenuate host antiviral responses [10]. Flavivirus NS3 (69 kDa) is the second largest viral protein (after NS5) and also the second most highly conserved viral protein among flaviviruses [11].

NS3 protein N-terminal domain contains a serine protease (peptidase_S7), whereas its C-terminal region contains NTPase and RNA helicase activities (Flavi_DEAD and HELICc, respectively); both domains are often present in a variety of helicases and helicase-related proteins [12]. NS3 protein N-terminal domain peptidase_S7 accompanies cofactor NS2B to form an NS2B–NS3 complex [13], playing a part in cleaving the polyprotein [14]. The C-terminus of NS3 involves three different enzymatic activities: RTPase, NTPase, and helicase [15]. RTPase cleaves a phosphoric hydrogen bond of 5’-triphosphorylated RNA [16], which is generally recognized as the first step of the three sequential enzymatic reactions of RNA 5’-capping; this is essential in viral translation and RNA stability [17]. The enzymatic hydrolysis of NTPase can supply energy to power the virus translocation and unwinding process [18]. Current research demonstrated that the dengue virus NS3hel is capable of establishing an ATP-dependent stable equilibrium between RNA unwinding [19] and annealing [20], allowing the modulation of the two antithetical activities of this enzyme through ATP concentration [21,22]. In West Nile virus, the hydrophilic N-terminal portion of NS4A acts as a cofactor facilitating NS3hel to maintain the unwinding rate of viral replication on ATP deficiency by anchoring the complex into the endoplasmic reticulum [23]. Some researchers supposed that NS3hel may be involved in uncoiling secondary structures of genomic RNA, displaying transacting protein cofactors [24], and separating the transiently formed double-stranded RNA (dsRNA) intermediates in the course of polymerization reaction catalyzed by NS5 RdRP [25].

Yeast two-hybrid technology has been widely used in recent years as a platform for discovering the interaction between proteins and proteins in living cells [26], which serve as a technical base for our study work [27]. The yeast two-hybrid system is performed in yeast to explore protein interactions; even weak, transient effects between proteins can also be detected sensitively by reporter gene products [28,29].

In this study, we intended to find potential binding partners of interest between NS3 protein and host cell protein using a yeast two-hybrid system and conduct verification in virtue of yeast regressive validation and GST Pull-Down assay [30]. In the follow-up study, we investigated the transcription and expression changes of the interacting protein and the phosphorylation level of relevant signaling pathway proteins after transfection. Our results put forward a new theory for the innate immune escape of DTMUV.

## 2. Materials and Methods

### 2.1. Y2H Assays

#### 2.1.1. Bait Yeast Strain Preparation

Referring to NS3 of the DTMUV sequence database on the National Center for Biotechnology Information, one pair of polymerase chain reaction (PCR) primers is designed to amplify the full-length NS3 gene. Viral RNA was extracted from liver tissue of infected duck through a TRIzol RNA Extraction Kit (Tiangen Biotech, Beijing, China). RNA was reverse transcribed into first-strand cDNA according to the manufacturer’s protocols using FastKing RT Kit (Tiangen Biotech). Amplification of specific NS3 fragment requires the use of PCR technology via TransTaq DNA Polymerase High Fidelity Kit (TransGen Biotech, Beijing, China). Reverse transcription-PCR (RT-PCR) products were analyzed by 1% agarose gel electrophoresis and purified (Omega Bio-tek, Guangzhou, China) and then cloned into a linearized pGBKT7 cloning vector using ClonExpress II One-Step Cloning Kit (Vazyme Biotech, Nanjing, China). The recombinant pGBKT7-NS3 vector was extracted using TIANprep Plasmid Kit (Tiangen Biotech) and then digested to be verified by *Sma*I and *Sal*I.

Yeast competent cells were prepared using LiAc/PEG3350 chemical method by referring to the Yeastmaker Yeast Transformation System 2 User Manual (Takara, Dalian, China) using activated Y2H Gold yeast for plasmid transformation. pGBKT7-NS3 and pGBKT7-53 positive control were transferred to the competent cells and then incubated in YPD Plus and spread onto agar plates containing the appropriate SD selection medium for incubation at 30 °C until colonies appear. Yeast plasmid pGBKT7-NS3 was extracted using the Yeast Plasmid Mini Preparation Kit (Beyotime Biotech, Shanghai, China).

#### 2.1.2. Autoactivation and Toxicity Test

Y2H yeast strain transformed with the corresponding pGBKT7-NS3 was grown on SD/-Trp (SDO), SDO/X, and SDO/X/A and pGBKT7-53 positive control was grown on SD/-Trp/-Leu (DDO), DDO/X, and DDO/X/A, which were separately grown on different SD selected media for 3 to 7 days at 30 °C for the autoactivation and toxicity tests.

#### 2.1.3. Mating and Screening

A concentrated cultured bait strain (Y2H Gold (pGBKT7-NS3)) and a thawing library yeast strain (Y187 (pGADT7-duck embryo fibroblast (DEF) cDNA library)) were prepared. In accordance with the instructions introduced by the Matchmaker Gold Yeast Two-Hybrid System User Manual (Takara), the bait strain was blended with the Mate & Plate™ library of DEF, and then their mixture was cultured in 2× YPDA/Kan medium and incubated at 30 °C with slow shaking (30–50 rpm) for 20 to 24 h until typically three-lobed structures can be seen clearly under the microscope, and then spread and grown on different stringency selected SD media. Blue colonies were dissolved with Lysis Buffer for Microorganism to Direct PCR (Takara) with sequencing primers of 5AD and pGAL4. Plasmids inserted with a length of less than 500 bp were abandoned, and the rest of the plasmids were extracted from blue colonies. All positive interactions must be subjected to further analysis to detect duplications and verify them to be genuine.

### 2.2. Regressive Verification

Three specific primers were designed for different domains S7, DEXDc, and HELICc to amplify separate gene fragments to recombine with pGBKT7. Meanwhile, peroxiredoxin 1 (PRDX1) was amplified using a specific primer to recombine with pGADT7. Prey plasmid pGADT7-PRDX1 was co-transferred with the corresponding bait plasmid into Y2H system to undertake regressive verification on SD/-Trp/-Leu/His/Ade/X/A (QDO/X/A) at 30 °C for 3 to 5 days. Five groups were equally divided for verification. First, because the subsequent experiments were performed in HEK293 cells, to avoid differences in some key binding sites of PRDX1, both duck and human PRDX1 were verified with full-length NS3. Next, duck PRDX1 was verified with domains S7, DEXDc, and HELICc, with the purpose of finding the specific domain that may interact with PRDX1.

### 2.3. Laser Scanning Confocal Microscopy Observation

HA-tagged eukaryotic expression vector pCAGGS-HELICc was constructed and co-transfected into HEK293 cells together with eukaryotic green fluorescent protein (GFP)-tagged eukaryotic expression plasmid pEGFP-PRDX1. After 24 h, transfection efficiency was observed, and the specific expression of the target protein was detected by western blot analysis. After fixation and permeabilization, the HA-labeled primary antibody and the Alexa 594 fluorescent secondary antibody were sequentially incubated for 1.5 h. Cells were subjected to 4’,6-diamidino-2-phenylindole (DAPI) nuclear staining for 5 min and finally observed under a confocal microscope.

### 2.4. Intracellular Reactive Oxygen Species (ROS) Detection

HEK293 cells were first transfected with pCAGGS and pCAGGS-PRDX1. When PRDX1 was highly expressed for about 24 h, cells were infected with DTMUV and treated for 6 h, whereas the Rosup positive control group was set in parallel. Finally, the probe of DCFH-DA was diluted at 1:1000 and treated at 30 °C for 20 min referring to the ROS Assay Kit (MKBio, Shanghai, China). Cells were then observed under a fluorescent inverted microscope immediately.

### 2.5. Glutathione S-transferase (GST) Pull-Down Assay

GST-tagged prokaryotic expression vector pGEX-6p-1-HELICc and GFP-tagged eukaryotic expression vector pEGFP-PRDX1 were constructed. The expression of proteins was determined by western blot analysis. Following the manual of Pierce GST Protein Interaction Pull-Down Kit (Thermo Scientific, Shanghai, China), first, the prepared GST-HELICc bait protein was incubated for 6 h for immobilization on glutathione agarose. Then, prey protein PRDX1 was passed through the column and finally captured by the immobilized GST-tagged bait protein. Next, the bait-prey complex protein of GST-HELICc-PRDX1-GFP was eluted by glutathione elution buffer. Finally, the protein was validated by western blot analysis using the monoclonal antibody (mAb) of GFP and GST tag. This experiment was repeated to ensure that the results are credible.

### 2.6. Real-time Polymerase Chain Reaction (PCR) and Phosphorylation Level of p38

pCAGGS-HELICc and pEGFP-PRDX1 were transfected into HEK293 cells separately and cultivated for 48 h and 72 h. RNA was extracted from HEK293 cells using RNAprep Pure Cell Kit (Tiangen Biotech). qPCR analysis was performed using ABI PRISM 7700 Sequence Detection System (Perkin–Elmer Applied Biosystems) with One-Step PrimeScript RT-PCR Kit (Takara) according to the manufacturer’s protocols to measure the mRNA expression of PRDX1 and p38. Relative expression values were normalized to the internal control of GADPH. Next, western blot analysis was performed on cells transfected with HELICc and co-transfected with HELICc and PRDX1 using p38 (T180+Y182) phosphorylated antibody.

## 3. Results

### 3.1. Construction of the Recombinant Plasmid

**Figure 1:**
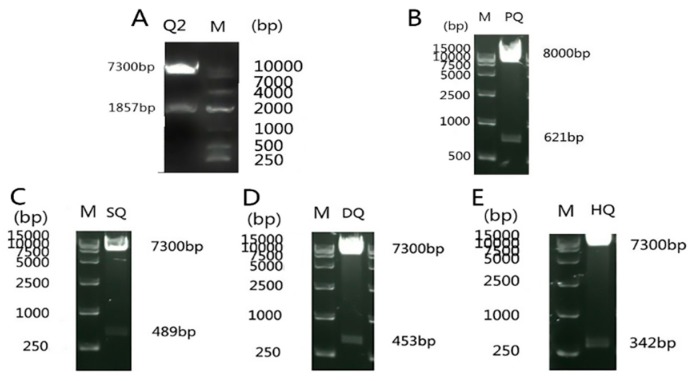
Identification of double enzyme digestion of pGBKT7-NS3, pGADT7-PRDX1, pGBKT7-S7, pGBKT7-DEXDc, and pGBKT7-HELICc: (**A**) bait plasmid pGBKT7-NS3, (**B**) prey plasmid pGADT7-PRDX1, (**C**) bait plasmid pGBKT7-S7, (**D**) bait plasmid pGBKT7-DEXDc, and (**E**) bait plasmid pGBKT7-HELICc.

PCR amplification primers were designed for different gene fragments, and the underlined part of primers is the homologous section of vectors (Table 1). The positive recombinant plasmid pGBKT7-NS3 was identified by *Sma*I and *Sal*I incision enzyme digestion, which display 7.3 and 1.9 kb fragments, respectively, as expected (Figure 1A). Positive recombinant prey plasmid pGADT7-PRDX1 and three-domain bait plasmid pGBKT7-S7/DEXDc/HELICc were identified by *Eco*RI and *Bam*HI incision enzyme digestion, which displays 621, 489, 453, and 342 bp gene fragments, respectively (Figure 1B–E).

### 3.2. Testing Autoactivation and Toxicity

**Figure 2:**
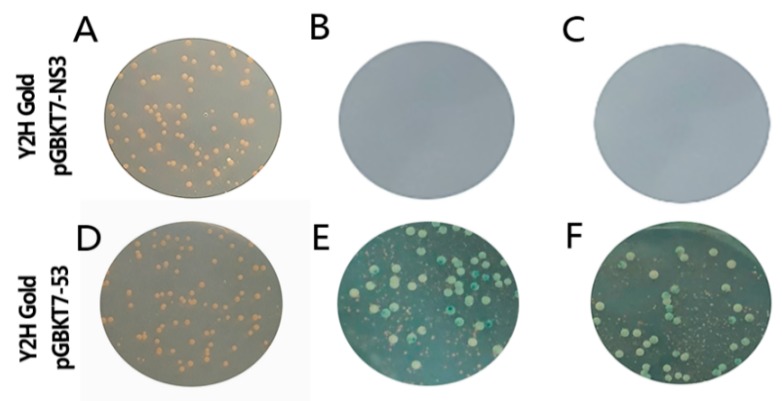
Autoactivation and toxicity verification of NS3 bait protein: (**A**–**C**) Y2H yeast transformed with bait pGBKT7-NS3 grow on SDO, SDO/X, and SDO/X/A culture media and (**D**–**F**) Y2H yeast transformed with pGBKT7-53 positive control grow on DDO, DDO/X, and DDO/X/A culture media.

As illustrated in Fig. 2, the Y2H yeast transformed with pGBKT7-53 positive control all grew well on DDO, DDO/X, and DDO/X/A culture media, and blue clones appeared in DDO/X and DDO/X/A (Figure 2E,F). Y2H yeast transformed with the bait pGBKT7-NS3 can grow white colony on SDO (Figure 2A), and there was no significant difference in the number and morphology of yeast colonies compared with the control group, indicating that it has no inhibitory effect on the growth of yeast (Figure 2A,D). However, it cannot grow on SDO/X and SDO/X/A, thus showing that it is unable to activate downstream reporter gene expression (Figure 2B,C). Therefore, it was confirmed that the constructed bait plasmid pGBKT7-NS3 has no toxicity and self-activating effects on Y2H and subsequent screening experiments can be performed.

### 3.3. Screening of Mate & Plate Library

**Figure 3:**
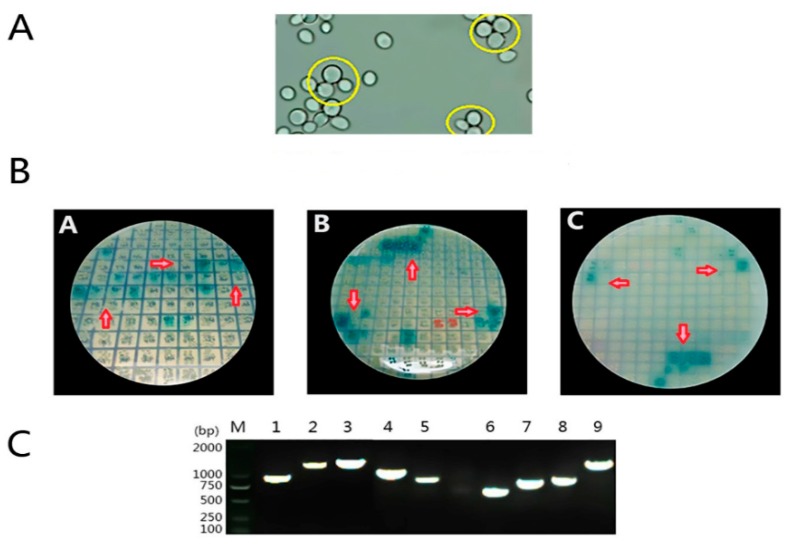
High-stringency screening by the yeast two-hybrid system. (**A**) Observation of zygote three-lobed structures under the microscope. (**B**) Positive blue colonies grow on QDO/X/A nutrient-deficient medium. (**C**) Amplification results of the positive yeast. M, marker 2000; 1–9, amplification of DEF cDNA library gene fragments 700 to 1500 bp in length.

After 20 h mating, a zygote that has a three-lobed structure typically displays a shape similar to Mickey Mouse‘s face, which can be seen under a phase-contrast microscope, showing that the bait and library plasmid coexist in a heterozygous diploid cell at this time (Figure 3A). Nearly 60 blue colonies selected from these plates were inoculated on high-stringency QDO/X/A medium (Figure 3B). PCR results of the yeast lysate showed that the size of the obtained nine fragments was about 700 to 1500 bp (Figure 3C). The fragments aligned with the database show that the result has repeatability. The nine proteins encoded by the gene are MGST1, ERCC4, WIF1, WDR75, ACBD3, PRDX1, RPS7, ND5, and LDHA; their biological functions are indicated in Table 2.

### 3.4. RNA-seq Analysis of Duck Tembusu Virus (DTMUV)-infected Cells

**Figure 4:**
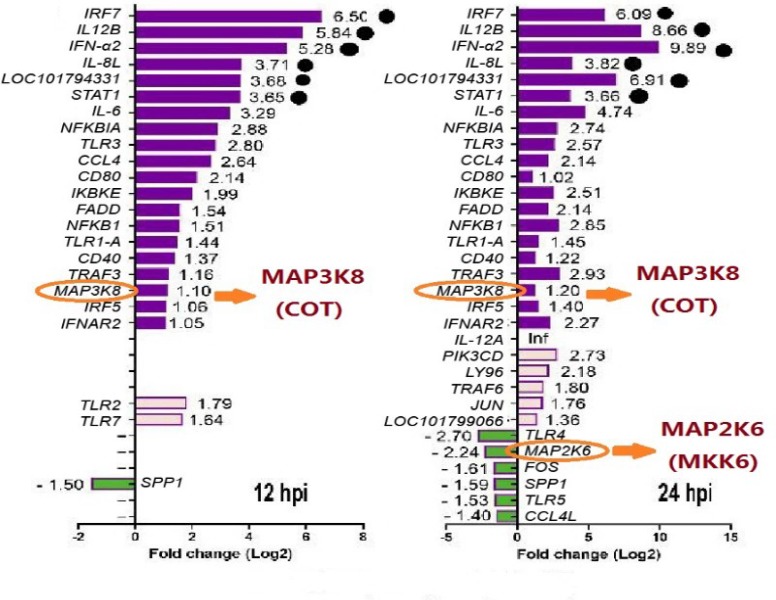
RNA-seq results of the Toll-like receptor (TLR) signaling pathway. Mitogen-activated protein kinase (MAP3K8) (COT) showed up-regulation of transcription levels after 12 h and 24 h infection of duck Tembusu virus (DTMUV), whereas MAP2K6 (MKK6) was down-regulated only after 24 h.

Based on the analysis of differential gene expression of the Toll-like receptor (TLR) pathway in the RNA-seq results measured in the laboratory, some reference data related to the mitogen-activated protein kinase (MAPK) pathway were obtained. It can be seen that both MAP3K8 (COT) and MAP2K6 (MKK6) present significant differential expression; those kinases are key components in the MAPK cascade signaling pathway, providing a reference for the identification of PRDX1 host protein and the subsequent study of oxidative stress (OS)-activated p38/MAPK pathway (Figure 4).

### 3.5. Intracellular Reactive Oxygen Species (ROS) Level Detection

**Figure 5:**
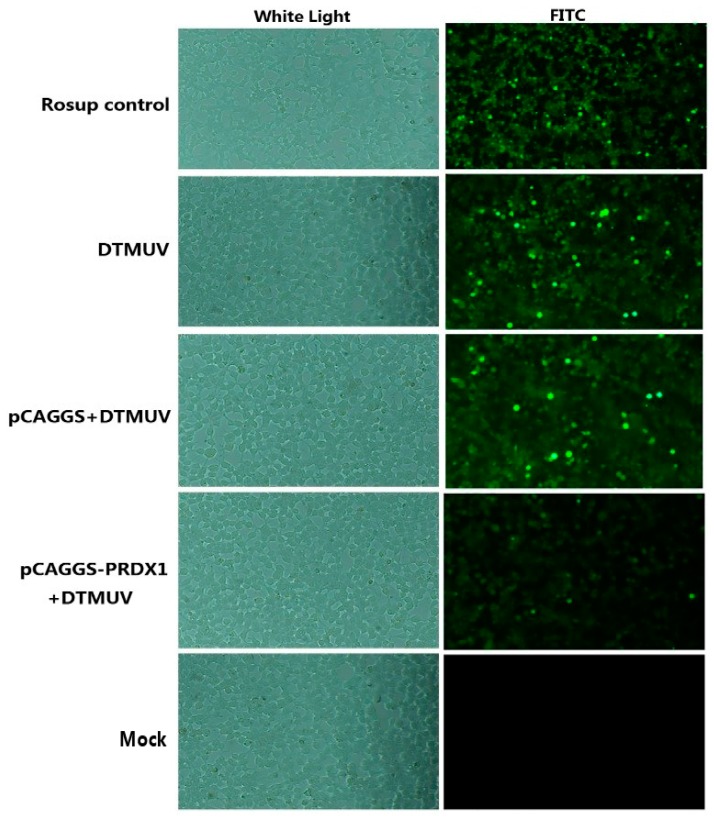
Determination of intracellular ROS levels. Rosup control, positive control; duck Tembusu virus (DTMUV), DTMUV infection; pCAGGS + DTMUV, pCAGGS-HA transfection and DTMUV infection; pCAGGS-PRDX1 + DTMUV, pCAGGS-PRDX1 transfection and DTMUV infection; Mock, without any transfection and DTMUV infection.

According to the ROS assay kit, cells in different treatment groups were observed under white light and fluorescein isothiocyanate (FITC). There was a strong fluorescence signal in the Rosup control, and it was also detected in all DTMUV-infected groups, which indicated that the ROS of the intracellular environment can be presented in a high-level stage after the virus invades the cells. At the same time, the fluorescence degree of the PRDX1 group was significantly weaker than that of the empty plasmid transfection group, indicating that PRDX1 can significantly reduce the intracellular ROS level (Figure 5).

### 3.6. Regressive Verification

**Figure 6:**
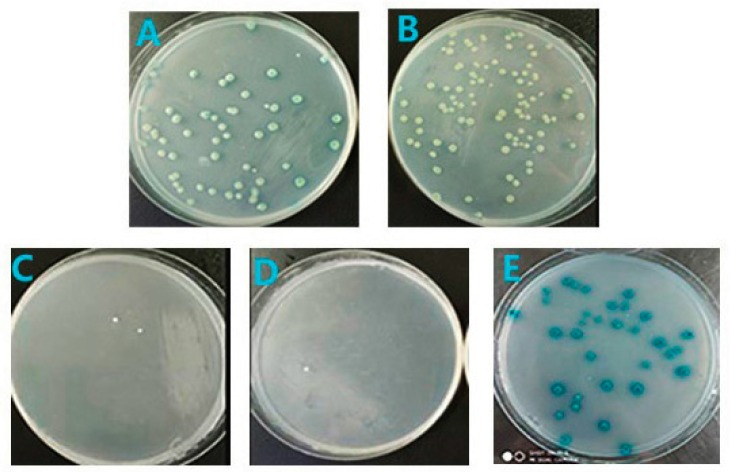
Regressive verification of NS3 and its three domains S7/DEXDc/HELICc with PRDX1: (**A**) full-length NS3 versus duck PRDX1 growing on QDO/X/A, (**B**) full-length NS3 versus human PRDX1 growing on QDO/X/A, (**C**) S7 versus duck PRDX1 growing on QDO/X/A, (**D**) DEXDc versus duck PRDX1 growing on QDO/X/A, and (**E**) HELICc versus duck PRDX1 growing on QDO/X/A.

Results show that blue clones are able to grow on Figure 6A,B,E but not on Figure 6C,D, which demonstrated that HELICc may be an interaction-specific domain in which the two are combined and PRDX1 interacts with the HELICc domain of NS3. At the same time, human PRDX1 may have similar interaction sites with duck PRDX1, which can interact with NS3 in the same way (Figure 6A,B).

### 3.7. Co-Localization of PRDX1 and HELICc

**Figure 7:**
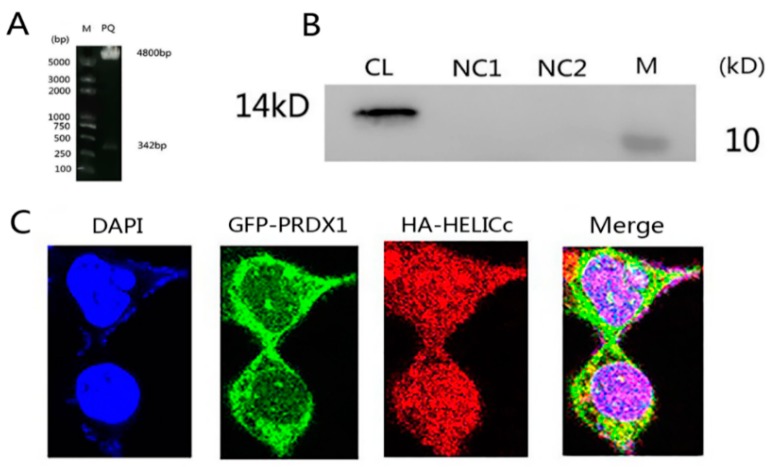
(**A**) Laser confocal intracellular co-localization of HELICc and PRDX1. Enzyme digestion of pCAGGS-HELICc. (**B**) Western blot analysis of HELICc. CL, lysate of HA-HELICc transformed cells; NC1 and NC2, protein of HA empty plasmid; M, protein marker. (**C**) Laser confocal observation. DAPI, nuclear location; GFP-PRDX1, PRDX1 cellular localization; HA-HELICc, HELICc cellular localization; Merge, PRDX1 and HELICc cellular co-localization.

Positive recombinant plasmid pCAGGS-HELICc was identified by incision enzyme digestion, which display 4.8 and 342 bp fragments, respectively, as expected (Figure 7A). Western blot analysis showed the prokaryotic expression of HA-HELICc fusion protein with a molecular weight of about 14 kDa (Figure 7B). Three excitation light channels were selected for DAPI, GFP, and Alexa 594 for three-dimensional laser scanning, showing that PRDX1 is localized in the cytoplasm and HELICc is mainly distributed in the area around the nucleus of the cytoplasm. Eventually, an overlapped image is generated, showing that PRDX1 and HELICc do indeed co-localize in a certain area within the cytoplasm of the cell, with the possibility of interaction (Figure 7C).

### 3.8. Glutathione S-transferase (GST) Pull-Down Assay

**Figure 8:**
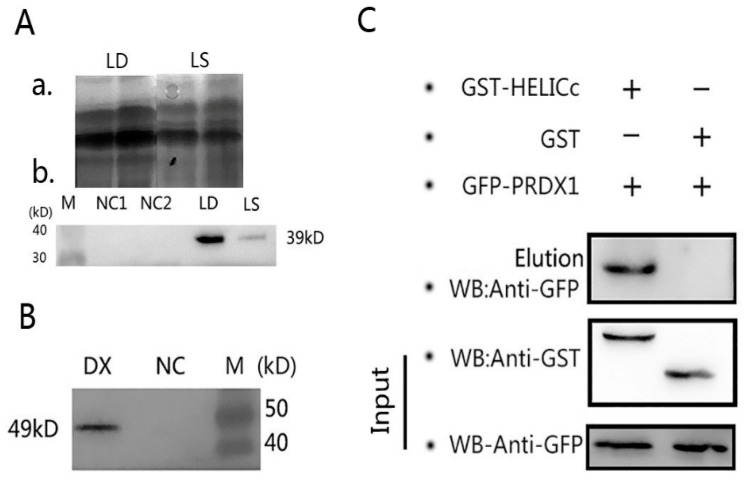
(**A**) GST Pull-Down assay of HELICc and PRDX1. Sodium dodecyl sulfate-polyacrylamide gel electrophoresis (SDS-PAGE) results of GST-tagged recombinant protein GST-HELICc in inclusion body protein (LD) and soluble protein (LS; a) and western blot analysis of GST-HELICc protein in inclusion body protein and soluble protein (b). (**B**) Western blot analysis of GFP-tagged recombinant protein GFP-PRDX1 (DX). (**C**) Verification of interaction between HELICc and PRDX1 by GST Pull-Down assay. Input, anti-GFP; GFP-PRDX1 using GFP-tagged mouse mAb; Anti-GST, GST-HELICc, and GST using GST-tagged mouse mAb; Elution, using both GFP and GST-tagged mAb.

To ensure the correct natural folding structure of the protein, sodium dodecyl sulfate-polyacrylamide gel electrophoresis (SDS-PAGE) results showed that GST-HELICc was expressed as a soluble protein after optimizing the expression conditions, but the expression level was lower than that of the inclusion body protein (Figure 8A). Western blot analysis showed the prokaryotic expression of GST-HELICc fusion protein with a molecular weight of about 39 kDa including a GST tag of about 25 kDa (Figure 8A) and the eukaryotic expression of GFP-PRDX1 fusion protein of about 49 kDa including a GFP tag of about 26 kDa (Figure 6B). Two specific lines of GST-HELICc and GFP-PRDX1 were detected in the GSH eluate but no line with GFP tag in the control group, which indicated that indeed an interaction exists between the two proteins of HELICc and PRDX1 (Figure 8C).

### 3.9. Real-Time Polymerase Chain Reaction (PCR) and p38 Phosphorylation Analysis

**Figure 9:**
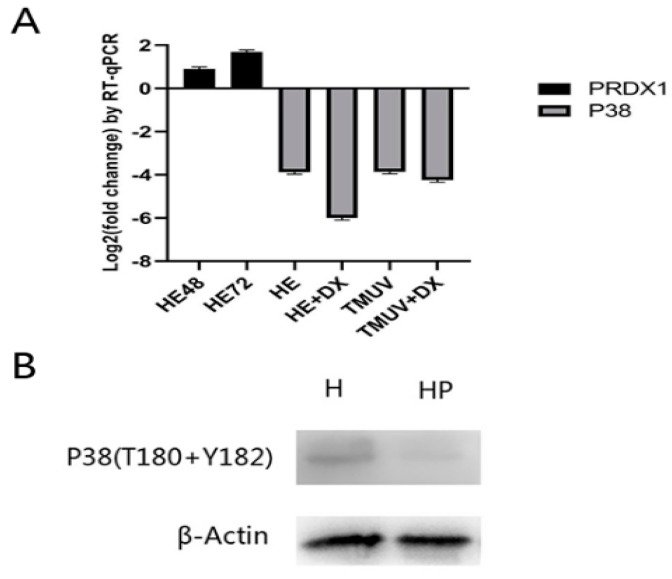
Analysis of quantitative polymerase chain reaction (PCR) and phosphorylation level of p38. (**A**) Quantitative PCR analysis of PRDX1 and p38. X-axis: HE48 and HE72, HELICc transfected 48 and 72 h; HE, HELICc transfected 72 h; HE+DX, PRDX1 and HELICc co- transfected 72 h; TMUV, DTMUV infection; TMUV+DX, DTMUV infection and PRDX1 transfected 72 h. Y-axis: log2(fold change) by quantitative RT-PCR. (**B**) Western blot analysis of the phosphorylation level of p38. H, HELICc transfected 72 h; HP, HELICc, and PRDX1 co- transfected 72 h; P38(T180 + Y182), Thr180, and Tyr182 phospho-p38 rabbit mAb; β-Actin: β-actin rabbit mAb.

Real-time PCR results in all the groups were statistically analyzed compared to the expression levels of empty pCAGGS-HA and pEGFP-C1, showing that a significant up-regulation of the mRNA transcription level of PRDX1 was observed at 48 h and 72 h in the HELICc transfection group. Later, we measured the transcription level of p38, showing a significant down-regulation in the treatment with HELICc and DTMUV, indicating that up-regulation of PRDX1 in these two groups affected p38 transcription. Meanwhile, the results showed that the mRNA transcription level of p38 with high expression of PRDX1 was reduced more than before, indicating that PRDX1 can actually decrease the transcription of p38 (Figure 9A). The phosphorylation of p38 in co-transfected HELICc and PRDX1 was significantly lower than that in the transfected HELICc alone using western blot analysis, suggesting that overexpression of PRDX1 can reduce the phosphorylation level of p38, thereby inhibiting the p38/MAPK pathway (Figure 9B). Referring to the previous results, the increase of ROS levels in DTMUV infection may cause intracellular OS and activate the p38/MAPK pathway to induce apoptosis. HELICc now can reduce p38 phosphorylation and intracellular ROS levels by targeting the up-regulation of PRDX1. On the one hand, it can inhibit the p38/MAPK pathway; on the other hand, it can inhibit the occurrence of OS-mediated apoptosis.

## 4. Discussion

In this study, a yeast two-hybrid system was applied to screen potential binding protein partners of DTMUV non-structural protein NS3 in DEF. Seven host proteins were discerned eventually. Protein NS3, as a multifunctional domain, implements further exploration to figure out which domain of S7/DEXDc/HELICc interacts with the target, of which the most interesting host protein is ascertained for further yeast two-hybrid regression to confirm the specificity and authenticity of the interaction. According to the DTMUV-infected RNA-seq result, the validated target protein, together with other pending proteins, extends to in-depth research toward viral life cycle regulation, viral immune escape, and apoptotic signaling pathways after viral infection of host cells.

There are two problems in the application of the yeast two-hybrid screening system: one is that there are more false-positives and the other is low transformation efficiency [31]. False-positives are mainly due to the fact that the bait protein itself is enabled to activate the reporter gene alone or the bait protein function is activated by another protein [32]. To eliminate the possibility of autonomous activation, a control group was set up and a self-activation test was performed [33], showing that our bait protein allows for subsequent screening steps. Low transformation efficiency is on account of the bait toxic effect on yeast growth or low abundance value of cDNA library [34]. In this experiment, the library titer is determined to overtop 1 × 10^7^ colony-forming units/ml, and a pre-experiment on the bait protein was conducted to affirm it [32]. To use a more efficient method to transfer the bait and library plasmids into different haploid yeasts Y2H and Y187, respectively, the bait and the prey are brought into the same zygotic diploid cells after hybridization, which enables an increase in transformation efficiency [35].

To obtain multiple high accuracy and repeatable positive results, we made improvements in the experimental procedure. In the screening of nutrient-deficient medium after hybridization, first we intended to get more phenotypic and well-grown yeast colonies cultivated on 60 DDO plates (150 mm); then, all yeast colonies were referred to one-to-one, plated on sterile paper and cultured in DDO/X/A and QDO/X/A with high-stringency screening. This stepwise screening procedure allowed us to obtain more positive clones.

Reviewing the biological functions of the candidate proteins and referring to ROS level increase and RNA-seq results after DTMUV infection, it was found that MGST1 and PRDX1 were correlated with intracellular OS and the p38/MAPK pathway, which provided a research direction for subsequent experiments. OS [36] refers to the disequilibrium of oxidation and anti-oxidation in the host [37], which is mainly characterized by the enrichment of a large number of oxidative intermediate free radicals [38]. Excessive ROS can cause damage to proteins, nucleic acids, and lipids, and even to cell membranes and organelles [39] on account of prolonged exposure to hydrogen peroxide and sulfhydryl alkylation molecules enriched in the cellular environment [40]. The oxidation–reduction reaction buffering mechanism present in cells can repair the oxidative damage in a phase of aerobic metabolism to a certain degree, mainly including superoxide dismutase, catalase, glutathione peroxide enzyme, and thioredoxin peroxidase (Prdx) [41,42].

The Prdx family possesses a function of purging ROS, reactive nitrogen species, and sulfur free radicals by means of this mechanism to protect cells from damage [43,44]. Prdx is prone to undergo peroxidation because its Cys51 exists as a thiolate anion during catalysis, whereas other cysteines (Cys17, Cys80, and Cys172) remain protonated in a neutral pH environment [45]. Prdx has high affinity with Trx disulfide, and the combination of the two acts as a substrate involved in a Trx peroxidase system that consists of NADPH, Trx, and TrxR [46,47]. In some cases, Prdx and Trx have been shown to act as a barrier to signal transduction [43]. In other cases, Prdx and Trx are sometimes involved in the transduction pathway as an active signal [48], regulating cell proliferation [49], differentiation, and apoptosis [50]. PRDX1 as a member of Prdx acts as a direct ROS level regulator molecule that regulates intracellular ROS-mediated OS levels.

The most basic and common responses to increased ROS are detoxification, increasing the level of repair enzymes and initiating apoptotic pathways in cells, thus successfully limiting the damage to the living organisms with the aid of these protective measures [47]. Many of these protective responses are mediated by the MAPK pathway, including extracellular signal-regulated kinase, c-Jun N-terminal kinase, and p38 [51]. Apoptosis signal-regulating kinase (ASK1) acts as a bridge and a linkage in the activation of the p38/MAPK signaling pathway [52,53]. Under OS, Trx1 primary binds to the N terminus of ASK1, and Trx1 is oxygenated and then dissociates from the Trx1–ASK1 complex [54]. An oligomeric form of ASK1, in which the threonine residue undergoes autophosphorylation performing kinase activity, activates p38 phosphorylation to mediate apoptosis [55]. PRDX1 in a high degree of affinity with Trx disulfide may likewise be involved in the aforementioned activation pathway as an effective signal [56].

NS3 HELICc has an ATP-binding site capable of recruiting ATPase [57,58] using the mechanical energy supplied from ATP hydrolysis to exert the function of unwinding RNA duplex [58], promoting the reaction of intermolecular annealing, and assisting in the mutual conversion of dsRNA and single-stranded RNA to establish a stable state between RNA structures [59]. This process requires a large amount of ATP to provide energy, and that increases the workload of the mitochondria [60], discharging a large amount of ROS enriched during the stage of mitochondrial aerobic respiration. In this case, OS may occur, leading to mitochondrial dysfunction, organelle membrane damage, and even apoptosis, which ultimately hinder the viral life cycle activity [61].

We hypothesized that PRDX1 in the redox buffer system can be recruited to the unwinding region through binding to specific sites in HELICc domain. In this way, it may regulate the level of ROS and participates in mediating the apoptotic pathway of p38/MAPK induced by OS, enabling the virus to complete its life cycle activities, thus suggesting a new mechanism for virus evasion of innate immunity.

## Figures and Tables

**Table 1:** Primers for construction of recombinant plasmids.

Gene	Sense Primer Sequence	Antisense Primer Sequence
pGBKT7- NS3	ATGGCCATGGAGGCCGAATTC GGGAGGAGTCATCTGGGAT	TGCGGCCGCTGCAGGTCGACG TCTCTTTCCACTCGCAAA
pGBKT7- S7	ATGGCCATGGAGGCCGAATTC GAAAGGAAGAGAGCCGAAG	TGCGGCCGCTGCAGGTCGAC CTTCTTCTTCCTTCTTCTC
pGBKT7-DEXDc	ATGGCCATGGAGGCCGAATTC GAAAACATGCTGCGAAAAA	TGCGGCCGCTGCAGGTCGAC CCGAGTTGGAGTCCGGAAA
pGBKT7-HELICc	ATGGCCATGGAGGCCGAATTC ATAACAGACTTTCAAGGAA	TGCGGCCGCTGCAGGTCGAC ATCCCTTCCAACCCGTCCA
pGADT7-PRDX1	CCAGTGAATTCCACCCGGGT ATGTCTTCAGGAAAGGCTT	AGCTCGAGCTCGATGGATCCC GTTTCCACTCGGACTGAA
pEGFP- PRDX1	TCAAGCTTCGAATTCTGAC TCGTTGCAACAAATTGATGA	ATCTAGATCCGGTGGATCC TGGGCACTTCTGCTTGGAGA
pCAGGS- PRDX1	TTCGAGCTCATCCATGGTACC ATGTCTTCAGGAAAGGCTT	ATTAAGATCTGCTAGCTCGAG GTTTCCACTCGGACTGAA
pCAGGS- HELICc	TTCGAGCTCATCCATGGTACC ATAACAGACTTTCAAGGA	ATTAAGATCTGCTAGCTCGAG ATCCCTTCCAACCCGTCC
pGEX-6P-1-HELICc	TCCAGGGGCCCCTGGGATCC ATAACAGACTTTCAAGGAA	GCCGCTCGAGTCGACCCGGGAA ATCCCTTCCAACCCGTC

**Table 2:** The information of the gene products interacting with NS3 according to Y2H system screening.

Gene Annotation	Fragment Size (bp)	Repetition	Protein Function
Microsomal glutathione S-transferase 1 (MGST1)	465	3	Conjugation of reduced glutathione to a wide number of exogenous and endogenous hydrophobic electrophiles.
DNA repair endonuclease (ERCC4)	2742	1	Catalytic component of a structure-specific DNA repair endonuclease responsible for the 5-prime incision during DNA repair.
Wnt inhibitory factor 1 (WIF-1)	1140	1	Binds to WNT proteins and inhibits their activities.
WD repeat-containing protein 75 (WDR75)	2493	1	Ribosome biogenesis factor. Involved in nucleolar processing of pre-18S ribosomal RNA.
Peroxiredoxin-1 (PRDX1)	582	4	Plays a role in cell protection against Oxidative Stress by detoxifying peroxides and as a sensor of hydrogen peroxide-mediated signaling events.
Required for rRNA maturation (RPS7)	597	2	Required for rRNA maturation
Golgi resident protein GCP60 (ACBD3)	486	3	Involved in the maintenance of Golgi structure by interacting with giantin, affecting protein transport between the endoplasmic reticulum and Golgi
NADH-ubiquinone oxidoreductase chain 5 (ND5)	1824	3	Core subunit of the mitochondrial membrane respiratory chain NADH dehydrogenase that is believed to belong to the minimal assembly required for catalysis.
l-lactate dehydrogenase A chain (LDHA)	999	1	Catalytic activity
